# Early optimal parenteral nutrition and metabolic acidosis in very preterm infants

**DOI:** 10.1371/journal.pone.0186936

**Published:** 2017-11-27

**Authors:** Francesco Bonsante, Jean-Bernard Gouyon, Pierre-Yves Robillard, Béatrice Gouyon, Silvia Iacobelli

**Affiliations:** 1 Réanimation Néonatale et Pédiatrique, Néonatologie, Centre Hospitalier Universitaire de la Réunion, Site Sud Saint Pierre, France; 2 Centre d’Etudes Périnatales de l’Océan Indien (CEPOI, EA 7388), Centre Hospitalier Universitaire de la Réunion, Site Sud Saint Pierre, France; Centre Hospitalier Universitaire Vaudois, FRANCE

## Abstract

**Background:**

It is currently recognized that an optimized nutritional approach, consisting of an early and substantial supply of protein and energy by parenteral route, may be beneficial for very low birth weight infants and recent guidelines endorse this strategy. However, the impact of the enhanced parenteral nutrition (PN) on acid-basic balance has never been investigated. The aim of the present study is to assess the effect of nutrient intake on acid-base homeostasis in a large population of preterm infants on PN.

**Methods:**

This observational study described the acid-base profile of very preterm infants (≤29 week’s gestation) receiving PN during the first week of life. For this purpose three different cohorts of infants who received increasing (group 1 to group 3) nutritional intakes were considered. Nutrition data were recorded daily and correlated to acid-base data (pH, base excess, and lactate). The outcome measure to assess metabolic acidosis was the base excess (BE).

**Results:**

161 infants were included. 1127 daily nutritional records and 795 blood gas data were analyzed. The three groups were different with regard to nutritional intravenous intakes. Group 3 in particular had a higher mean intake of both amino acids (3.3 ± 0.8 g/kg/d) and lipids (2.8 ± 1.4 g/kg/d) during the first week of life. Metabolic acidosis was more severe in the group with the highest parenteral intake of amino acids and lipids: mean BE = -8.7 ± 3.4 (group 3); -6.4 ± 3.4 (group 2); -5.1 ± 3.0 (group 1)]. At the multivariate analysis the significant risk factors for metabolic acidosis were: gestational age, initial base excess, amino acid and lipid intravenous intakes.

**Discussion:**

Acid-base homeostasis was influenced by the nutritional intake. Earlier and higher intravenous amino acid and lipid intakes particularly increased the risk of metabolic acidosis. The nutritional tolerance was different depending on gestational age, and the smaller infants (24–26 week’s gestation) displayed greater acidotic disequilibrium and a higher need of bicarbonate.

## Introduction

Over the last decades, the approach to nutrition of the premature infant during early life has been characterized by the progressive increment of the recommended protein and energy intakes [1–3]. More recently, the advantages of ensuring early high amino acid and energy supply by the parenteral route have been confirmed in both very and extremely low birth weight infants. This allows the prevention of catabolism, boosting growth and avoiding extra uterine growth retardation [4–8]. In parallel, the possible side effects of this nutritional approach were not always carefully investigated in terms of biological impact. Several of our own articles and those of other authors highlighted some important effects on electrolyte balance that were initially not foreseen, such as hypokalemia, hypophosphatemia and hypercalcemia [9–12].

Nowadays, little is known about the possible effects of enhanced nutrition on early metabolic balance and acid-base homeostasis.

In the past, intravenous mixtures containing synthetic amino acids were found to lead to metabolic acidosis in infants [13], but this effect was not observed with the use of the newer solutions specifically designed for premature infants (Trophamine®, Primène®) [14, 15].

However, the impact on acid-basic balance of the recent parenteral nutrition (PN) with higher and earlier amino acid and energy intakes has never been specifically investigated in large cohort studies.

In 2015 the ESPGHAN, ESPEN, ESPR and CSPEN societies discussed and reached consensus for the updating the neonatal and pediatric parenteral nutrition guidelines issued in 2005) (ESPGHAN 2005) and the updated recommendations will be published in 2017. One of us (SI) participated in the design and the finalization of the new guidelines and several changes were applied in our neonatal intensive care unit, as early as June 2015. The main changes in our practice affected the immediate starting of lipid emulsions from birth and the rapid increment of both amino acid and lipid intravenous intakes from postnatal day 2 onwards.

This allowed us to evaluate the impact of the new nutritional approach on acid-base balance during the first week of life, and to compare it with two previous cohorts receiving lower nutritional intakes according to foregoing recommendations.

The aim of the present study is to assess the effect of nutrient supply on acid-base homeostasis in a large population of preterm infants on total or partial PN.

## Methods

### Design and study population

This was an observational study comparing three cohorts of infants which received different and progressively increasing nutritional intakes by PN during the first week of life.

The study periods for the 3 cohorts were the following: 1 June 2006 to 30 September 2007 (Group 1), 1 January 2014 to 31 March 2015 (Group 2) and 1 June 2015 to 31 May 2016 (Group 3).

All consecutive infants born at less than 30 weeks of gestational age during each study period were included in the analysis. Patients were excluded if they died within the first week of life, if clinical data were incomplete, or if blood gas analysis from day 2 to day 7 of life was missing.

### Ethics statement

This study was conducted in accordance with French legislation. As per new French law applicable to trials involving human subjects (Jardé Act), a specific approval of an ethics committee (“comité de protection des personnes”- CPP) is not required for this non-interventional study based on retrospective, anonymized data of authorized collections and written parental consent is not needed.

### Data collection

Nutrition data of group 1 were available according to the collection strategy previous described and authorized [11].

Nutrition data of the groups 2 and 3 were recorded by the unit computer prescriber ordering entry system (Logipren®) where all data are stored and available for prescription practices studies [16], as authorized by the National Data Protection and Privacy Commission (authorization number 1854394) since 2014.

Similarly, the administration of buffer (sodium bicarbonate) as treatment for acidosis was available for all the study population.

For the three groups, all enteral and parenteral nutrient intakes were recorded daily for the first 7 days of life and were expressed as a daily mean of the first week of life.

Sodium and chloride inputs were also recorded, and the differential Na^+^–Cl^-^ mean intake was calculated as this is known to influence metabolic acid-basic abnormalities [17, 18].

Data on acid-base balance (pH, base excess and lactate) were available when collected for previous studies [11] or were obtained from infant clinical files and recorded at birth and for the first 7 days of life.

The outcome measure to assess acidosis (metabolic acidosis) was the base excess (BE). BE measures at birth or during the first hours of life were considered to be representative of prenatal or perinatal period and were defined as “baseline values”. So, only BE measures from day 2 to day 7 were used in order to evaluate the impact of nutrition on acid-basic balance. Then BE measures were corrected for the administration of bicarbonate in the previous 24 hours by de following formula:

$$CorrectedBE=\frac{BE-3.3\;*\;HCO3}{Kg}$$

(HCO_3_: mmol of bicarbonate intravenous infusion during the 24 previous hours).

All records of acidosis with high lactate levels (blood lactate > 4 mmol/L) were excluded from the analysis, as possibly due to hypoxia—hypoperfusion mechanism.

In order to study the occurrence of acidosis according to the PN administration, the nutritional intakes of the previous 24 hours were taken into account.

Finally the estimated buffer need (mmol/kg/week) during the first week of life was calculated by the formula:

$$BufferNeed=\frac{BE\min\;*\;0.3+CumulHCO3}{Kg}$$

(BEmin: minimal base excess day 2 to 7, Cumul HCO_3_: cumulative weekly intravenous bicarbonate infusion in mmol).

### Growth measures

Weight was recorded at birth and at 36 weeks of gestation (or at hospital discharge if before 36 weeks of postmenstrual age—PMA). Weight centiles and Z-scores for weight (means and standard deviation for GA) were calculated according to Fenton curves [19]. Delta Z-score of weight between birth and 36 weeks was also calculated. Intrauterine growth restriction was defined as weight <10^th^ centile at birth. Extrauterine growth restriction was defined as weight <10^th^ centile at 36 weeks in infants with normal weight at birth.

### Statistical analysis

Continuous variables were presented as means ± standard deviations. Normality was checked using the Shapiro-Wilk test. Comparisons among the three groups were performed using ANOVA or Kruskal-Wallis tests when appropriate.

To validate the independent association of nutritional intakes with the BE concentration we realized a multiple regression linear analysis. The model used all variables associated with BE at a p-level <0.10 at the univariate analysis, as well as all potentially confounding factors associated with metabolic acidosis according to the literature (i.e.: differential Na^+^–Cl^-^ intake).

A p-value below 0.05 was considered significant. All analyses were performed using MedCalc software (version 12.3.0; MedCalc Software's, Ostend, Belgium).

## Results

A total of 161 children were included into the 3 groups and 1127 daily records were available for analysis. Nutritional intakes were available for all the daily records and blood gas analysis data for 795 of them (71%), 142 at birth and 653 between day 2 and day 7 of life.

The three groups were similar for gestational age and weight at birth and they were significantly different with regard to nutritional intakes and postnatal growth (Table 1 and Table 2).

**Table 1:** Characteristics and postnatal growth in the 3 groups.

	Group 1 (N = 58)	Group 2 (N = 50)	Group 3 (N = 53)	P ANOVA
**Gestational Age, weeks**	**27.8 ± 1.2**	**27.8 ± 1.6**	**27.5 ± 1.6**	**0.39** ^**¤**^
**Birth Weight, grams**	**1018 ± 201**	**960 ± 202**	**930 ± 244**	**0.10**
**Birth Weight Z-score**	**-0.42 ± 0.75**	**-0.57 ± 0.79**	**-0.62 ± 0.80**	**0.17**
***Growth data***				
**Weight Z-score at 36 weeks of PMA**	**-1.39 ± 0.72**	-0.83 ± 0.79*	-0.88 ± 0.76*	**<0.001**
**Delta Z-score**	**-0.97 ± 0.69**	-0.26 ± 0.68*	-0.27 ± 0.79*	**<0.001**
**IUGR (Birth Weight <10°centile), %**	**14**	**17**	**15**	**0.90**
**Catch-up growth at 36 weeks of PMA, %**	**2**	**5**	**7**	**0.41**
**EUGR at 36 weeks of PMA, %**	**45**	17*	20*	**0.003**
**Total GR at 36 weeks of PMA, %**	**57**	29*	28*	**0.003**

IUGR: Intrauterine Growth Restriction. EUGR: Extrauterine Growth Restriction. GR: Growth Restriction. Data are expressed as mean ± SD.

*P<0.05 vs. group 1.

^¤^Kruskal-Wallis test.

**Table 2:** Nutrient intakes for the 3 groups.

	Group 1 (N = 58)	Group 2 (N = 50)	Group 3 (N = 53)	P ANOVA
***Mean daily intakes (1***^***st***^ ***week)***				
**Amino acids, g/kg/d**	**2.1 ± 0.8**	3.1 ± 0.7*	3.3 ± 0.8*	**<0.001** ^**¤**^
**Lipids, g/kg/d**	**1.6 ± 1.2**	**1.8 ± 1.2**	2.8 ± 1.4*^§^	**<0.001** ^**¤**^
**Carbohydrates, g/kg/d**	**10.2 ± 2.8**	**10.4 ± 2.4**	11.6 ± 3.0*^§^	**<0.001** ^**¤**^
**Chloride, mmol/kg/d**	**2.8 ± 2.0**	1.5 ± 1.0*	**2.5 ± 1.4**^**§**^	**<0.001** ^**¤**^
**Sodium, mmol/kg/d**	**2.6 ± 1.9**	2.1 ± 1.1*	**3.0 ± 1.2** ^**§**^	**<0.001** ^**¤**^
**Differential Na-Cl intake, mmol/kg/d**	**-0.1 ± 0.7**	0.6 ± 0.7*	0.5 ± 0.9*	**<0.001** ^**¤**^
**IV amino acids g/kg/d**	**2.0 ± 0.8**	2.9 ± 0.6*	3.1 ± 0.7*	**<0.001** ^**¤**^
**IV lipids, g/kg/d**	**1.2 ± 0.9**	**1.3 ± 1.1**	1.9 ± 1.1*^§^	**<0.001** ^**¤**^
** IV carbohydrates g/kg/d**	**9.4 ± 2.8**	**10.4 ± 2.4**	11.6 ± 3.0*^§^	**0.005** ^**¤**^
**Enteral nutrition, ml/kg/d**	**12 ± 25**	**14 ± 23**	20 ± 30*	**0.01** ^**¤**^
**Enteral amino acids g/kg/d**	**0.1 ± 0.3**	0.2 ± 0.3*	0.2 ± 0.4*	**<0.001** ^**¤**^
**Enteral lipids, g/kg/d**	**0.4 ± 0.9**	**0.5 ± 0.9**	0.8 ± 1.0*^§^	**<0.001** ^**¤**^
***Amino acid intake*, *g/kg/d***				
**Day 1**	**1.0 ± 0.2**	**2.0 ± 0.1**	**2.0 ± 0.3**	
**Day 2**	**1.4 ± 0.2**	**2.5 ± 0.3**	**2.6 ± 0.4**	
**Day 3**	**1.8 ± 0.4**	**2.9 ± 0.4**	**3.2 ± 0.5**	
**Day 4**	**2.2 ± 0.4**	**3.3 ± 0.3**	**3.6 ± 0.4**	
**Day 5**	**2.6 ± 0.4**	**3.5 ± 0.4**	**3.9 ± 0.3**	
**Day 6**	**2.8 ± 0.5**	**3.7 ± 0.4**	**4.0 ± 0.2**	
**Day 7**	**2.9 ± 0.8**	**3.7 ± 0.5**	**4.0 ± 0.2**	
***Lipid intake*, *g/kg/d***				
**Day 1**	**0.0 ± 0.1**	**0.2 ± 0.3**	**0.7 ± 0.5**	
**Day 2**	**0.6 ± 0.3**	**1.0 ± 0.6**	**1.6 ± 0.4**	
**Day 3**	**1.1 ± 0.4**	**1.5 ± 0.7**	**2.3 ± 0.7**	
**Day 4**	**1.5 ± 0.5**	**2.0 ± 0.8**	**3.0 ± 0.6**	
**Day 5**	**2.0 ± 0.6**	**2.4 ± 0.8**	**3.5 ± 0.7**	
**Day 6**	**2.7 ± 0.8**	**2.8 ± 0.8**	**4.0 ± 0.8**	
**Day 7**	**3.1 ± 0.9**	**3.1 ± 0.8**	**4.3 ± 0.9**	
***Glucose intake*, *g/kg/d***				
**Day 1**	**6.5 ± 0.8**	**7.2 ± 0.6**	**7.2 ± 0.6**	
**Day 2**	**7.5 ± 0.5**	**8.2 ± 0.9**	**8.7 ± 1.2**	
**Day 3**	**8.8 ± 0.9**	**9.4 ± 1.1**	**10.4 ± 1.3**	
**Day 4**	**10.4 ± 1.1**	**10.8 ± 1.2**	**12.0 ± 1.5**	
**Day 5**	**11.8 ± 1.2**	**11.8 ± 1.3**	**13.4 ± 1.5**	
**Day 6**	**13.0 ± 1.6**	**12.8 ± 1.4**	**14.5 ± 1.3**	
**Day 7**	**13.5 ± 1.8**	**13.2 ± 1.6**	**15.1 ± 1.4**	

IV: intravenous. Data are expressed as mean ± SD.

*P<0.05 vs. group 1.

^**§**^ P<0.05 vs. group 2.

^¤^Kruskal-Wallis test.

Carbohydrate and lipid intakes were higher in Group 3 and amino acid intakes in both Group 2 and 3 compared to Group 1. These differences were mainly due to parenteral nutrition and in a lesser portion to the enteral one. Table 2 shows in detail the daily intake for each nutriment.

**Fig 1:**
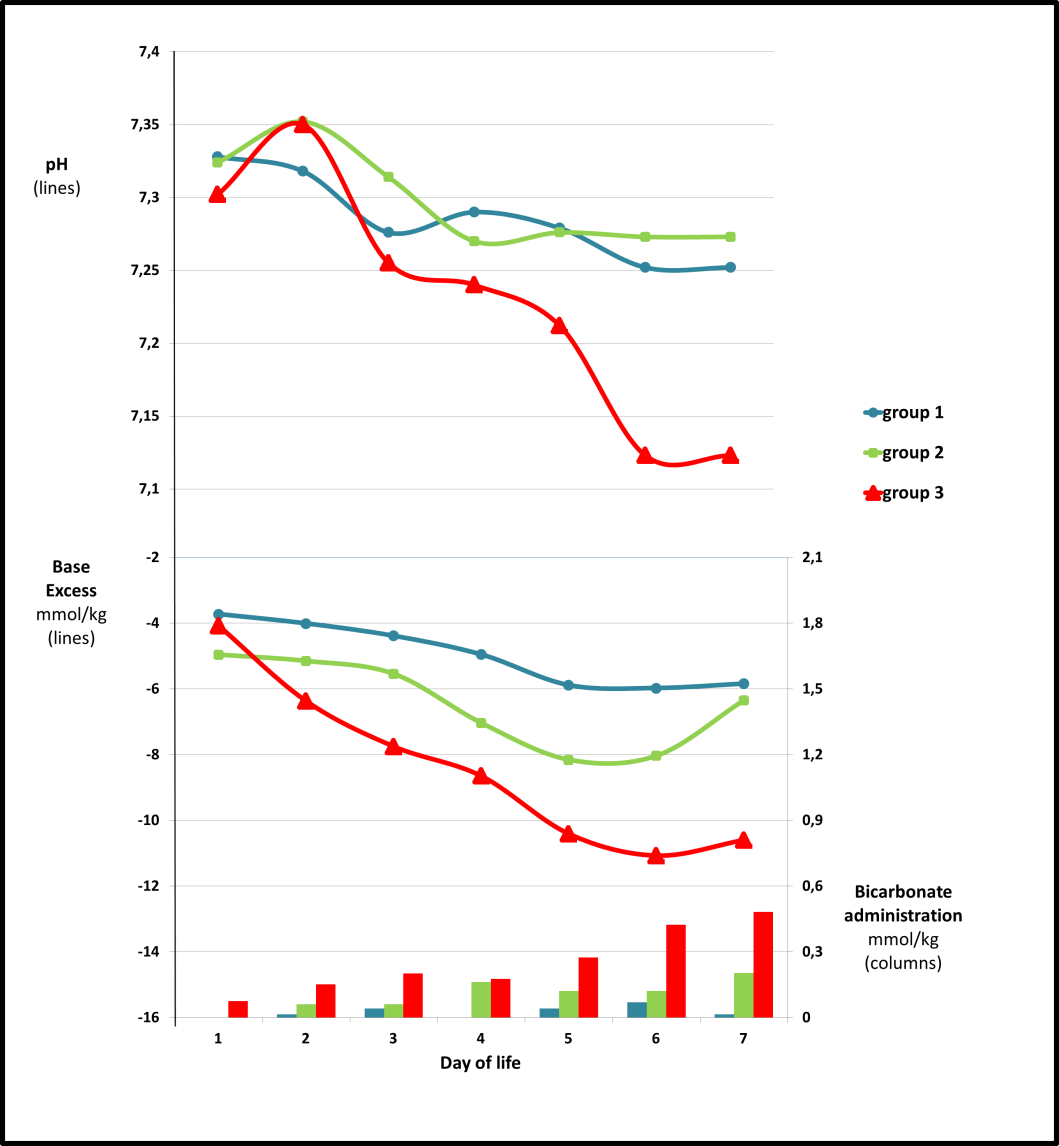
pH and base excess in the first week of life. The figure shows the trend for mean pH and base excess (uncorrected) in the three groups during the first week of life. Bicarbonate administration is also shown.

Table 3 shows a higher rate of metabolic acidosis during the first week of life in Group 3, compared to Group 2 and in the Group 2 compared to Group 1. It also shows the corresponding estimated buffer deficit in the three groups. Fig 1 illustrates the trends in pH and BE during the first week within the 3 groups and the need for buffer solution administration.

**Table 3:** Acid-base data for the 3 groups.

	Group 1 (N = 58)	Group 2 (N = 50)	Group 3 (N = 53)	P ANOVA
***Acid-base data***				
**pH at birth**	**7.33 ± 0.06**	7.32 ± 0.10*	7.30 ± 0.10*	**0.33**
**Base excess at birth**	**-3.7 ± 3.4**	-4.9 ± 3.7*	-4.4 ± 3.3*	**0.27**
**Lactate at birth, mmol/L**	**2.8 ± 2.0**	**3.7 ± 2.5**	**3.5 ± 2.3**	**0.60** ^**¤**^
**pH at day 2–7**	**7.28 ± 0.08**	**7.30 ± 0.10**	7.24 ± 0.13*^§^	**<0.001**
**Base excess at day 2–7 (uncorrected)**	**-5.1 ± 3.0**	-6.4 ± 3.4*	-8.7 ± 3.4*^§^	**<0.001**
**Lactate at day 2–7**	**1.5 ± 0.9**	**1.6 ± 0.9**	**1.8 ± 1.3**	**0.69** ^**¤**^
**Bicarbonate infusion, mmol/kg/d**	**0.03 ± 0.16**	**0.10 ± 0.39**	0.25 ± 0.64*	**<0.001** ^**¤**^
***Estimated Buffer Need for the 1***^***st***^ ***week (mmol/Kg)***				
**Infants 24–26 weeks**	**2.2 ± 2.9**	5.8 ± 2.7*	9.1 ± 4.3*^§^	**<0.001** ^**¤**^
**Infants 27–29 weeks**	**2.6 ± 2.1**	3.0 ± 2.5*	4.7 ± 3.0*^§^	**<0.001** ^**¤**^

Data are expressed as mean ± SD.

*P<0.05 vs. group 1.

^**§**^ P<0.05 vs. group 2.

^¤^Kruskal-Wallis test.

**Fig 2:**
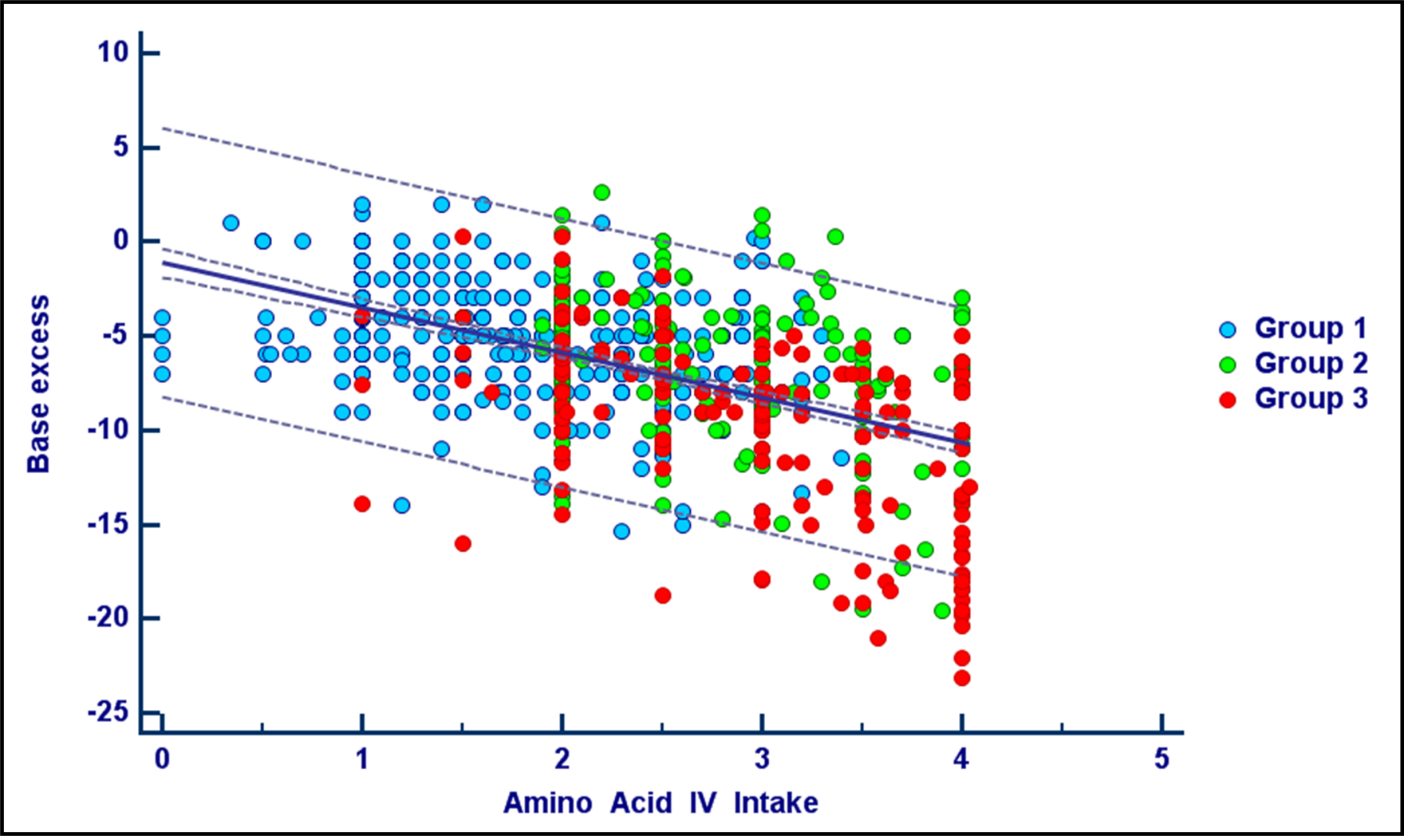
Amino acid intakes and base excess. The figure shows the scatter diagram and regression line between daily amino acid intravenous intakes and base excess. Amino acid intake was considered from one day before and expressed as g/kg/d. Base excess was corrected for the administration of bicarbonate. Records with lactate > 4 mmol/L were excluded. Correlation coefficient r = 0.51, P<0.001.

**Fig 3:**
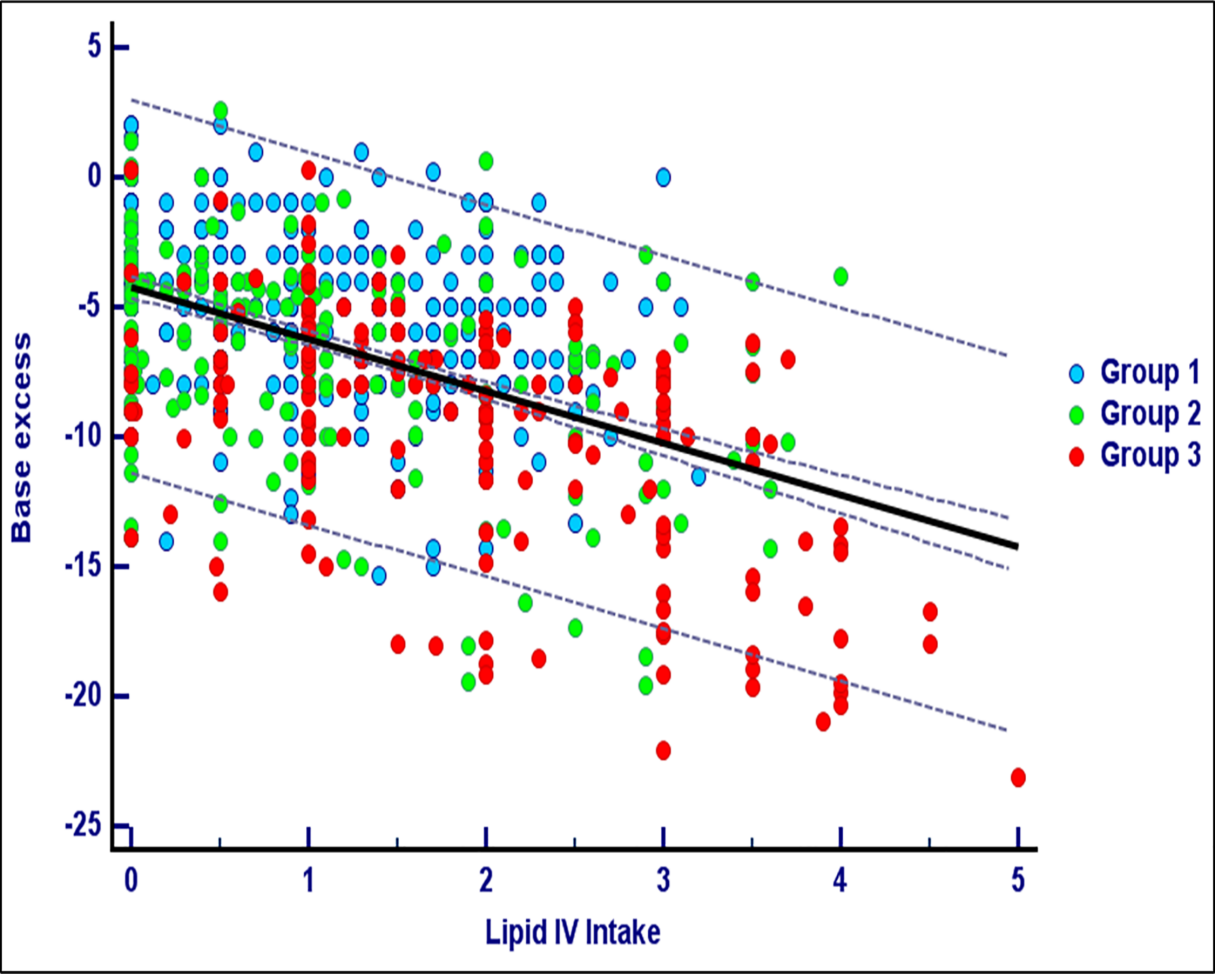
Lipid intakes and base excess. The figure shows the scatter diagram and the regression line between the daily lipid intravenous intakes and the base excess. Lipid intake was considered from one day before and expressed as g/kg/d. Base excess was corrected for the administration of bicarbonate. Records with lactate > 4 mmol/L were excluded. Correlation coefficient r = 0.50, P<0.001.

The relationship between the amino acid and lipid intravenous intakes with BE is shown in Figs 2 and 3. In both analyses the association with BE was found to be significant for each of the three groups (linear regression procedure, data not shown).

Table 4 shows the results of the multivariate analysis for metabolic acidosis (BE) which confirms the significant association of intravenous amino acid and lipid intakes as independent risk factors.

**Table 4:** Multivariate analysis for base excess (BE).

Multiple Linear Regression for Base excess				
(general R^2^ = 0.45)				
	coefficient	R^2^ partial	t	P
**Gestational Age**	**1.04**	**0.42**	**12.0**	**<0.0001**
**Initial BE**	**0.22**	**0.24**	**6.3**	**<0.0001**
**Lipid IV intake**	**-1.08**	**-0.22**	**-5.9**	**<0.0001**
**Amino acid IV intake**	**-0.84**	**-0.15**	**-3.8**	**0.0001**
***Variables not included in the final model***				
**Differential Na-Cl intake**				
**Carbohydrates IV intake**				
**Day of life**				

A multiple linear regression model was performed using 672 base excess records from day 2 to 7 of life. Base excess was corrected for the administration of bicarbonate. Nutrition intakes were considered from one day before. Records with lactate > 4mmol/L were excluded (N = 13, 1.9%)

**Fig 4:**
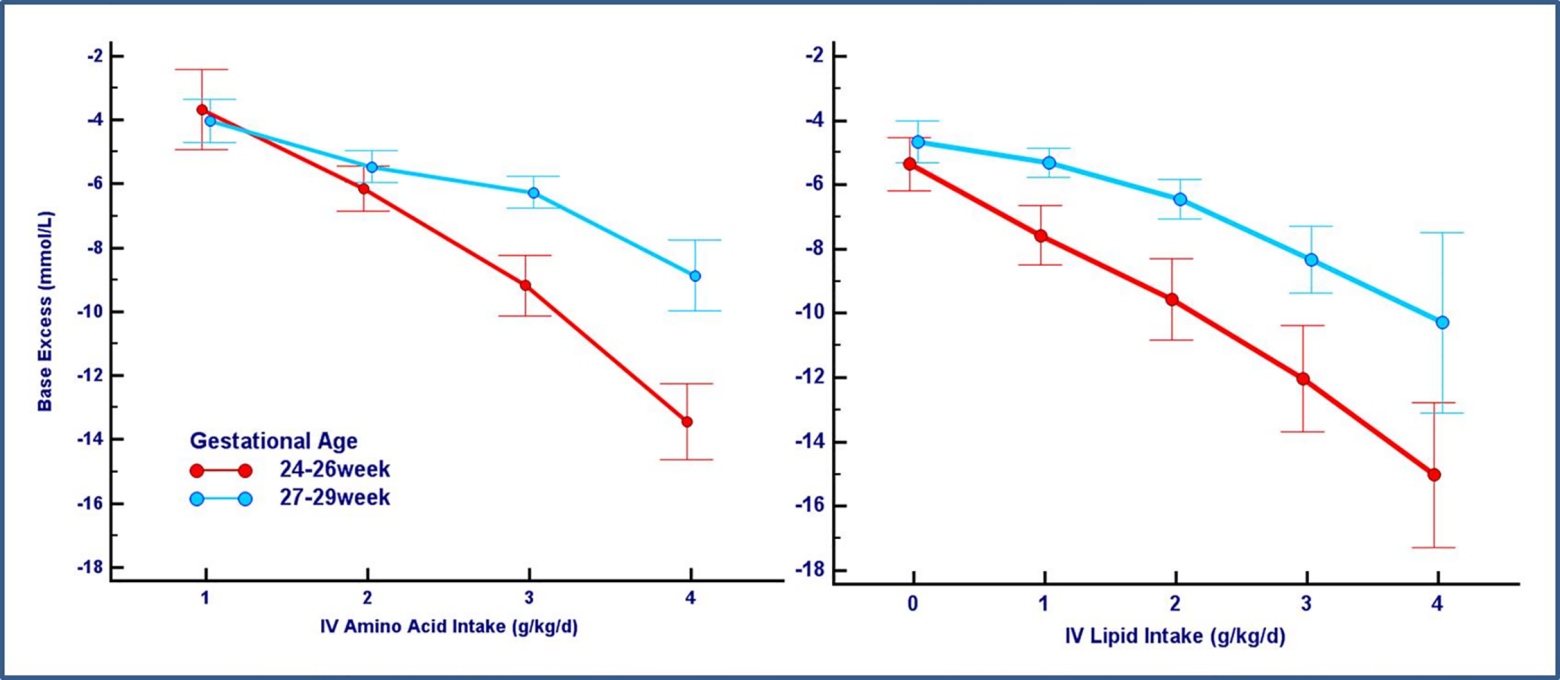
Influence of gestational age. The figure shows a different impact of parenteral intake of amino acids and lipids on base excess levels according to gestational age categories.

Fig 4 displays the different trend of BE in children of different gestational age and according to increasing intakes of parenteral amino acids and lipids. Extreme premature babies (born less than 24–26 weeks of gestation) showed a poor metabolic tolerance to high parenteral intake of nutrients and a greater tendency to acidosis.

## Discussion

The main finding of our study is that the enhanced and early nutritional supply according to the new recommendations exposes very premature babies to substantial acid-base disequilibrium during the first week of life, in the form of a non-lactic metabolic acidosis. The proportion of this imbalance directly related to the nutrition seems to be important and may be clinically relevant, considering the achieved pH levels and the need for buffer solution administration in this population.

Few investigations have addressed the impact on acid-base homeostasis of early and “aggressive” administration of parenteral amino acids to low birth weight infants, but their study design and target population were different to ours. Jadhav et al. described acid-base status in premature infants receiving parenteral amino acids at 1.5g/kg/d, or 3g/kg/d for a short (5h), extended (24h) or for prolonged (3–5 days) duration and they concluded that metabolic acidosis was independent of amino acid dose between 2 to 5 days after birth, and mostly associated with co-morbidities [20]. Differently from us, they defined acidosis with a cut off (pH<7.25), they studied older infants (less than 32 weeks and 1850g) and they did not take into account any other nutritional intakes than amino acids. The review from Guellec et al. emphasized that new recommendations for PN could be associated with metabolic acidosis via some previously described mechanisms, i.e.: the increase in the amino acid ion gap, hyperchloremic acidosis, and ammonia acidosis [21].

For the first time our study draws the attention to the importance of both amino acid and lipid intakes as the most involved risk factors for metabolic acidosis during PN. The higher intravenous intake and the rapidity of the increase do play a role in the occurrence and in the severity of metabolic acidosis. Interesting also, in Group 2 and 3 the rise of nutrient intakes promotes acidosis despite the optimization of sodium and chloride inputs performed in our unit. Indeed, the augmentation of differential Na^+^/Cl^-^ intake observed in groups 2 and 3 should play a protective role against acidosis [17].

The mechanisms involved in the increased rate of acidosis in infants exposed to enhanced PN cannot completely been elucidated in our cohort. Like adults, neonates must excrete acid generated by metabolism in the form of ammonia and titrable acid. So, the traditional explanation for metabolic acidosis (other than hyperchloremic) during PN in preterm infants has given emphasis to amino acids intolerance ahead of the low urinary excretion rate of ammonium [22] and the lower activity of key enzymes leading to ammonia production in infants [23]. Renal bicarbonate loss from the immature kidney may also play a role [24]. Of course all these mechanisms could be involved in our cohort, and our study had the limitation of not measuring blood urea levels or ammonium excretion. Beside this, we speculated that, in a more general way, a disproportionate acid body load may result from enhanced nutrition and may stress the immature metabolism of the very low birth weight infant. Acid bodies introduced by nutriments or from endogenous production (including those due to the possible ketogenic effect of lipid emulsions) may exceed the renal and hepatic metabolic ability and may be not tolerated in very preterm infants.

Another interesting issue addressed by our results is that the two other independent factors related to metabolic acidosis in the study population were the initial BE level, as shown by blood sample at birth or in the first hours of life, and—most importantly—the gestational age at birth. This effect was particularly evident in extremely preterm infants born at 24 to 26 weeks of gestation. In these babies the fall in pH and the base excess due to the increase of parenteral lipids and amino acids were more abrupt and sustained, and according to our experience it could persist beyond the first week of life. Moreover, these children often need parenteral nutrition support for a longer period, so they really deserve a particular attention to PN tolerance in clinical practice.

The main strengths of our study are the high quantity of nutritional and blood gas analysis data in a large population of very premature babies and the timeliness of data in relation to new nutritional recommendations. This report also has several limitations. Firstly, it is a non-randomized trial. Secondly, differences in periods and potential discrepancies in practice handling may exist among the cohorts. Thirdly, this study cannot conclude about the possible impact of early metabolic acidosis on VLBW infants’ outcome.

As reported by previous investigations [4–8], the enhanced and early nutritional supply, especially by intravenous amino acids, was able to sustain a better growth and to reduce the prevalence of extrauterine growth restriction in our population. However the interesting relationship between sustained acidosis and growth [24] could not be investigated here, according to the study design.

Even if due to these limitations no firm conclusion can be drawn based on our results, we believe that this study shed light on an important aspect of nutrition tolerance in very preterm infants.

This investigation cannot answer the question of how and whether we should face to metabolic acidosis related to PN. Based on our results, this issue warrants consideration at least for earlier gestational born infants and strategies focusing on prevention (through the reduction of nutritional intake or the adaptation of parenteral solutions) or treatment (established criteria for treating metabolic acidosis associated to PN) should be explored. Possibly a strategy of adapting the parenteral nutrition bags with buffer salts, as potassium lactate or sodium acetate, could be the most elegant solution and we have shown the calculated buffer need in Table 3.

New research is needed to confirm these findings and both randomized controlled trials and wide observational multicenter studies might be useful for this purpose.
